# Discriminative power of an adapted version of Nutritional Risk Screening 2002 applied to Brazilian older adults

**DOI:** 10.31744/einstein_journal/2020AO5309

**Published:** 2020-10-21

**Authors:** Midori Cabral Sugaya, Regiane Maio, Bruna Lúcia de Mendonça Soares, Cinthia Katiane Martins Calado, Glaucia Queiroz Morais, Ilma Kruze Grande de Arruda, Maria Goretti Pessoa de Araújo Burgos

**Affiliations:** 1 Universidade Federal de Pernambuco Recife PE Brazil Universidade Federal de Pernambuco, Recife, PE, Brazil.

**Keywords:** Aged, Nutritional status, Malnutrition, Nutritional assessment, Nutrition therapy

## Abstract

**Objective:**

To investigate the discriminative power of Nutritional Risk Screening 2002.

**Methods:**

A cross sectional study involving one hundred participants aged ≥60 years. The original and adapted versions of Nutritional Risk Screening 2002 and the Mini Nutritional Assessment were used. Nutritional Risk Screening 2002 adaptation consisted of a lower age cutoff (60 years or older) for addition of one extra point to the final score.

**Results:**

Screening using Nutritional Risk Screening 2002 revealed higher nutritional risk among patients aged ≥70 years (p=0.009), whereas screening using the adapted version of Nutritional Risk Screening 2002 revealed similar nutritional risk in both age groups (60-69 years and ≥70 years; p=0.117). Frequency of nutritional risk was highest when the Mini Nutritional Assessment was administered (52.7%), followed by the adapted and original versions of Nutritional Risk Screening 2002 (35.5% and 29.1%, respectively).

**Conclusion:**

The adapted version of Nutritional Risk Screening 2002 was more effective than the original version. However, further studies are needed to confirm these findings.

## INTRODUCTION

Nutritional risk is described as increased risk of morbidity and mortality due to inadequate nutritional status,^(^^1^^)^ often related to disease severity.^(^^2^^)^ Nutritional screening is used to detect nutritional risk and consists of a simple inquiry aimed to identify individuals requiring nutritional intervention to lower the risk of complications, infection and mortality,^(^^3^^)^ shorten hospital length of stay,^(^^4^^)^ improve quality of life and reduce healthcare costs.^(^^3^^)^ Nutritional intervention is required in individuals with nutritional risk^(^^2^^)^ to prevent malnutrition and resulting increased morbidity and mortality rates, with a worse prognosis for recovery from underlying diseases.^(^^3^^,^^5^^)^

The Nutritional Risk Screening 2002 (NRS 2002) questionnaire is a fast, user-friendly nutritional assessment tool,^(^^5^^–^^7^^)^ for detection of changes in nutritional status and determination of the need for interventions to maintain or recover adequate nutritional status in adults and older adults.^(^^8^^)^ However, NRS 2002 was derived from studies involving older adults aged 70 years and over, who receive 1 extra point in the final score as an additional risk factor.^(^^6^^)^ This assessment tool was developed in European countries, where older adults are defined as those aged 65 years and older. According to the World Health Organization (WHO), however, the cutoff should be set at a younger age in developing countries and older adults defined as those aged 60 years and older.^(^^8^^)^

Given the negative impact of malnutrition on older adults, studies aimed to adapt this screening method to the Brazilian older adult population are warranted, to promote early identification of those requiring nutritional intervention for adequate nutritional status maintenance or recovery.

## OBJECTIVE

To evaluate the discriminative power of an adapted version of the Nutritional Risk Screening 2002, in which the age cut-off for addition of one extra point to the final score was set at 60 years or more.

## METHODS

A cross-sectional study conducted to test the discriminative power of an adapted version of NRS 2002. Male and female patients aged 60 years and older and hospitalized in wards or surgical units of a university hospital in the city of Recife (PE), Brazil, participated in the study. Data collection was carried out between April and December 2016, and all participants signed an Informed Consent Form. Patients not eligible for anthropometric assessment, amputees, patients presenting with edema or ascites, transplanted patients, patients unable to provide information, and those undergoing enteral nutritional therapy were excluded.

Nutritional risk was assessed within 48 hours of admission using three structured questionnaires administered to each patient: original and adapted versions of NRS 2002 and the Mini Nutritional Assessment (MNA) – the last one is the gold standard.

The adapted version of NRS 2002 consisted of a modification of the original model proposed by Kondrup et al.,^(^^7^^)^ the difference being a lower age cutoff (60 years) for addition of 1 extra point in the final score. Nutritional risk classification was maintained, with a score of ≥3 considered indicative of risk.

The original version of NRS 2002 comprises two parts. The first addresses body mass index (BMI), weight loss, food intake changes and underlying disease severity. The second is limited to patients who meet one of first part criteria, and consists of deeper investigation and stratification of variables assessed in that part. At the end of the questionnaire, 1 point is added for individuals aged 70 years and older and nutritional risk defined as a score of ≥3 points.^(^^9^^)^

The MNA is thought to be the gold standard for nutritional assessment of older adults, and was created to investigate changes in body composition, recent changes in body weight, altered food intake patterns, degree of autonomy, and self-perception of health status in this population.^(^^3^^)^ This questionnaire is capable of predicting morbidity, mortality and clinical outcomes, and has 18 items divided into two parts. The first includes six screening items that generate a score of up to 14 points. Scores of 12 points or higher are defined as absence of nutritional risk, with no need to complete the rest of the questionnaire. Scores of 11 points or lower suggest nutritional risk or malnutrition and, in such cases, the questionnaire must be completed. Mini Nutritional Assessment includes anthropometric data, general assessment, diet and subjective assessment. Sum MNA scores allow the following nutritional risk classification: <17 points for malnourished, 17 to 23.5 points for risk of malnutrition, and ≥24 points for well nourished.^(^^10^^)^

Demographic and clinical variables were extracted from the patient records. Demographic variables corresponded to sex (male or female) and age (60 to 69 years or ≥70 years). Clinical variables included underlying disease upon admission (neoplasia, vascular disease, genitourinary, neurological or gastrointestinal disorders, problems such as fever, anorexia, liver disease, respiratory or ear-nose-throat conditions and presumptive diagnoses), and number of comorbidities (arterial hypertension, *diabetes mellitus* and chronic obstructive pulmonary disease) associated with underlying disease (none, one, or two or more comorbidities).

Anthropometric variables considered were BMI, arm circumference and calf circumference. Body mass index was defined as weight (kg) divided by height squared (m^2^). Weight (kg) was measured using an analytical scale (Filizola^®^, São Paulo, SP, Brazil; maximum capacity 150kg, accuracy 100g). Height was determined using an aluminum stadiometer mounted onto the scale in the horizontal Frankfurt plane,^(^^11^^)^ or using the Chumlea^(^^12^^)^ equation based on knee height. Body mass index cut-offs defined by Lipschitz^(^^13^^)^ were used for nutritional status classification.

Non-dominant arm circumference was measured using a non-elastic measuring tape (maximum length, 1.50m). Participants were instructed to flex the arm at a 90° angle for proper location of the midpoint between the acromion and olecranon. Participants were then instructed to extend the arm along the body with the palm facing the thigh. Arm circumference was measured at the aforementioned point, taking care to avoid skin compression and slack in the measuring tape.

Left calf circumference was measured at the widest point using a non-elastic measuring tape (maximum length, 1.50m). Patients were placed in the supine or sitting position with the knee flexed at a 90° angle.

Statistical Package for Social Science (SPSS), version 23.0 for Windows^®^, and Excel^®^ 2010 software were used in statistical analyses. Tests were applied with a 95% confidence interval (95%CI). Data were expressed as absolute frequencies and percentages. Associations between categorical variables were tested using the Pearson's χ^2^ test or the Fisher's exact (whenever the χ^2^ test was not applicable). Sensitivity, specificity, accuracy and positive and negative predictive values were determined for the original and adapted versions of NRS 2002 and compared to the MNA. The level of agreement between assessment tools was determined using Kappa statistics. The margin of error for statistical tests was set at 5% (p<0.05) and 95%CI were calculated.

This study was approved by the Human Research Ethics Committee of Center for Health Sciences of the *Universidade Federal de Pernambuco* (UFPE), protocol no. 1.488.532, CAAE: 52561116.0.0000.5208.

## RESULTS

The sample comprised 110 patients, most (63.6%) of them males. Just over half (53.6%) were aged 60 to 69 years, and 46.4% were aged 70 years or more (p=0.052). Reasons for hospitalization are listed in table 1, neoplasm and vascular disease being the most common (28.2% and 20.9%, respectively).

**Table 1:** Sample description (sex and clinical variables) according to age group of older adults admitted to a university hospital

Variable		Age group			p value*
		60 a 69 years	70 or more	Complete group	
Sex					0.857
	Male	38 (64.4)	32 (62.7)	70 (63.6)	
	Female	21 (35.6)	19 (37.3)	40 (36.4)	
Reasons for					0.052†
admisison					
	Neoplasm	16 (27.1)	15 (29.4)	31 (28.2)	
	Vascular disease	10 (16.9)	13 (25.5)	23 (20.9)	
	Genitourinary	4 (6.8)	10 (19.6)	14 (12.7)	
	Gastrointestinal	7 (11.9)	3 (5.9)	10 (9.1)	
	Neurological	3 (5.1)	4 (7.8)	7 (6.4)	
	Other‡	14 (23.7)	6 (11.8)	20 (18.2)	
Not informed		5 (8.5)	0	5 (4.5)	
	Comorbidity				0.372
	Nonexistent	23 (39.0)	23 (45.1)	43 (39.1)	
	One disease	20 (33.9)	9 (17.6)	25 (22.7)	
	Two or more	16 (27.1)	19 (37.3)	42 (38.2)	
	diseases				

Results expressed as n (%).

* Pearson's χ^2^ test;

† Fisher's exact test;

‡ other conditions: fever, anorexia, liver disease, respiratory disorders, ears-nose-throat problems, and presumptive diagnoses.

Nutritional risk estimates derived from the adapted version of NRS 2002 or MNA did not differ between age groups. In contrast, when the original version of NRS 2002 used, nutritional risk was more frequent among patients aged 70 years and older (p=0.009) (Table 2).

**Table 2:** Estimated nutritional risk using three different methods, according to age group in older adults admitted to a university hospital

Age group, years	Adapted NRS 2002		NRS 2002		MNA	
	n	p value	n	p value	n	p value*
60-69	17	0.117	11	0.009*	27	0.116
≥70	22		21		31	

* Pearson's χ^2^ test, p<0.05.

NRS: Nutritional Risk Screening 2002; MNA: Mini Nutritional Assessment.

Nutritional risk estimated using the adapted or original version of NRS 2002 was not significantly associated with diseases or comorbidities (Table 3). However, significant associations between nutritional risk and types of disease (p=0.019) and nutritional risk and comorbidities (p=0.022) were found when risk was estimated using MNA (data not shown in table).

**Table 3:** Associations between nutritional risk estimated using the adapted or original version of Nutritional Risk Screening 2002, and reasons for admission/ comorbidities in older adults admitted to a university hospital

Variable		Adapted NRS 2002			NRS 2002		
		Yes	No	p value	Yes	No	p value
Reason for hospitalization				0.419*			0.591*
Neoplasm		10 (32.3)	21 (67.7)		10 (32.3)	21 (67.7)	
	Vascular disease	10 (43.5)	13 (56.5)		7 (30.4)	16 (69.6)	
	Genitourinary	4 (28.6)	10 (71.4)		4 (28.6)	10 (71.4)	
	Gastrointestinal	3 (30.0)	7 (70.0)		2 (20.0)	8 (80.0)	
	Neurological	5 (71.4)	2 (28.6)		4 (57.1)	3 (42.9)	
Others†		6 (30.0)	14 (70.0)		4 (20.0)	16 (80.0)	
	Comorbidity			0.216			0.350
	None	18 (42.9)	24 (57.1)		12 (28.6)	30 (71.4)	
	One disease	11 (25.6)	32 (74.4)		10 (23.3)	33 (7.7)	
	Two or more diseases	10 (40.0)	15 (60.0)		10 (40.0)	15 (60.0)	

Results expressed as n (%).

* Pearson's χ^2^ test;

† Fisher's exact test.

NRS: Nutritional Risk Screening 2002.

Table 4 displays the frequency of nutritional risk according to MNA and the original or adapted version of NRS 2002 in patients aged 60 years and older. Nutritional risk frequency was highest when patients were submitted to MNA, followed by the adapted version of NRS 2002.

**Table 4:** Frequency of nutritional risk among patients aged 60 years or older (n=110) according to nutritional screening tool

Screening tool	Nutritional risk	No nutritional risk
MNA	52.7	47.3
Original NRS 2002	29.1	70.9
Adapted NRS 2002	35.5	64.5

Results expressed as %.

MNA: Mini nutritional assessment; NRS 2002: Nutritional Risk Screening 2002.

Table 5 displays agreement levels between methods, MNA being the gold standard. The adapted version of NRS 2002 was more sensitive than the original version (60.3% and 50% respectively), although still classified as insufficient. Summed sensitivity and specificity of the original and adapted versions of NRS 2002 corresponded to 144.2 and 152.6, respectively.

**Table 5:** Diagnostic accuracy and 95% confidence intervals of the adapted and original versions of Nutritional Risk Screening 2002 compared to Mini Nutritional Assessment (gold standard)

Instrument	Sensitivity	Specificity	PPV	NPV	Accuracy
	(n1/n)	(n1/n)	(n1/n)	(n1/n)	(n1/n)
Adapted NRS 2002	60.3 (35/58)	92.3 (48.52)	89.7 (35/39)	67.6 (48/71)	75.5 (83/110)
Original NRS 2002	50.0 (29/58)	94.2 (49.52)	90.6 (29/32)	62.8 (49/78)	70.9 (78/110)

PPV: positive predictive value; NPV: negative predictive value; NRS 2002: Nutritional Risk Screening 2002.

## DISCUSSION

Results obtained using the adapted version of NRS 2002 in this study are in keeping with findings derived from the original version reported in literature, *i.e*., higher specificity than sensitivity, suggesting adequate ability to detect patients without nutritional risk. In a study involving older adults based in Geneva and Berlin, Kyle et al.,^(^^14^^)^ reported a 28% rate of nutritional risk using NRS 2002. In that study, sensitivity, specificity, positive and negative predictive values (62%, 93%, 85% and 79%, respectively) were higher compared to those obtained with the Malnutrition Universal Screening Tool and reported in the NR Initiative.

However, as sensitivity increases the number of false positive results also increases. Likewise, higher specificity translates into higher numbers of false negative.^(^^15^^)^ Therefore, despite equivalent levels of agreement between MNA and both versions of NRS 2002 (moderate agreement; Kappa coefficient), the adapted version was thought to be descriptively more efficient due to higher sensitivity. Another positive impact of NRS 2002 adaptation was higher summed sensitivity and specificity compared to the original version – yet another factor supporting higher discriminative power of the adapted over the original version.

As to the main reasons for hospital admission, findings of this study reflect data described by Bezerra et al.,^(^^5^^)^ in a study conducted in a multi-specialty clinic located in the city of Natal (RN), in which most patients (26.9%) suffered from cancer, followed by genitourinary (11.5%), respiratory (11.5%) and gastrointestinal (7.7%) disorders. In contrast, musculoskeletal disorders (22.2%), respiratory (11.1%) and cardiovascular (11.1%) diseases were the major diagnoses at a private healthcare service located in the city of Porto Alegre (RS), as in developed countries.^(^^16^^)^ Indeed, Koren-Hakim et al.,^(^^17^^)^ reported hypertension (69.3%), cardiovascular disease (52.1%) and musculoskeletal disorders (42.3%) as major reasons for admission to hospital. Differences in sample characteristics may be explained by economic class disparities between study populations, given the association between higher purchasing power and better nutritional status.

In this study, no significant associations were found between disease and nutritional risk estimated using either version of NRS 2002. This may have been due to the fact that most patients were in the early stages of disease, and were admitted to hospital for underlying disease diagnosis. It should be noted that, unlike other nutritional screening tools, MNA does not account for underlying disease, which impacts determination of associations between reason for hospital admission and nutritional risk.

Nutritional risk frequency estimated using the original and adapted versions of NRS 2002 did not differ between patients suffering from neoplasm. Similar findings were reported by Lisboa da Silva et al.,^(^^18^^)^ in a sample comprising exclusively clinical patients, assessed using the original version of NRS 2002 at the same university hospital (nutritional risk rate among cancer patients, 38.4%).

Studies involving nutritional screening are based on different nutritional risk detection methods and measures, as well as patients with distinct diagnoses. Nutritional risk frequency in this study was lower compared to rates observed in a surgical ward (69.3%)^(^^19^^)^ and a medical clinic (51.3%) of a university hospital located in the city of Recife.^(^^18^^)^ This disparity may be explained by clinical specialties and disease-related differences between samples.

With regard to nutritional risk detection, rates derived from the adapted version of NRS 2002 and MNA were similar in both age groups (60-69 years and ≥70 years). In contrast, nutritional risk rates were significantly higher among individuals aged 70 years and older, when assessment was based on the original version of NRS 2002, suggesting this questionnaire is not a good screening tool for individuals aged 60 to 69 years.

Studies investigating agreement between NRS 2002 and MNA are scarce. In a study conducted by Neelemaat et al.,^(^^20^^)^ with older adults aged 60 years and older in the city of Amsterdam, Netherlands, NRS 2002 had numerically higher sensitivity (92%), specificity (83%), positive (70%) and negative (96%) predictive values compared to findings of this study. However, that study adopted anthropometric measures as the gold standard and these are not the best nutritional screening tool for older adults. Also, discriminative power of the method in this study was determined using Kappa statistics, which allows for more accurate comparison with the gold standard.

Limitations of this study must be accounted for. Sample size was too small to be representative. Moreover, given the cross-sectional design, outcomes are related to a specific time point, which precludes determination of potential cause-effect relations.

## CONCLUSION

Results of this study suggest that the adapted version of Nutritional Risk Screening 2002 has higher discriminative power in older adults aged 60 years or more compared to the original version, allowing for more effective detection of nutritional risk in hospitalized older adults. However, further studies are warranted to confirm this finding.
