# Supplementary material for: Discriminative power of an adapted version of Nutritional Risk Screening 2002 applied to Brazilian older adults
Source: Einstein (Sao Paulo). 2020 Oct 21;18:eAO5309. doi: 10.31744/einstein_journal/2020AO5309 (PMC8639137; doi:10.31744/einstein_journal/2020AO5309)
Supplement: Translation

# Translation: Poder diagnóstico discriminativo da versão adaptada do *Nutritional Risk Screening 2002* administrada em idosos brasileiros

**Authors:** Midori Cabral Sugaya, Regiane Maio, Bruna Lúcia de Mendonça Soares, Cinthia Katiane Martins Calado, Glaucia Queiroz Morais, Ilma Kruze Grande de Arruda, Maria Goretti Pessoa de Araújo Burgos

**Affiliations:** 1 Recife PE Brasil Universidade Federal de Pernambuco, Recife, PE, Brasil.

**Keywords:** Idoso, Estado nutricional, Desnutrição, Avaliação nutricional, Terapia nutricional

## Abstract

**Objetivo:**

Avaliar o poder de discriminação diagnóstica da ferramenta *Nutritional Risk Screening 2002*.

**Métodos:**

Estudo transversal com cem participantes com idade ≥60 anos. Foram aplicados o *Nutritional Risk Screening 2002* original, o *Nutritional Risk Screening 2002* adaptado e o *Mini Nutritional Assessment*. A adaptação do *Nutritional Risk Screening 2002* consistiu em diminuir o critério de idade, incluindo pontuação adicional para 60 anos de idade ou mais.

**Resultados:**

Maior risco nutricional ocorreu nos ≥70 anos quando aplicado o *Nutritional Risk Screening 2002* original (p=0,009), enquanto o *Nutritional Risk Screening 2002* adaptado apresentou risco nutricional semelhante em ambos os grupos (60-69 anos e ≥70 anos; p=0,117). A frequência de risco nutricional foi maior no *Mini Nutritional Assessment* (52,7%), seguido do *Nutritional Risk Screening 2002* adaptado (35,5%) e do *Nutritional Risk Screening 2002* original (29,1%).

**Conclusão:**

A adaptação do *Nutritional Risk Screening 2002* mostrou-se descritivamente mais eficaz do que a original, porém mais estudos devem ser realizados para confirmar os achados.

## INTRODUÇÃO

O risco nutricional é descrito como risco aumentado de morbidade e mortalidade devido ao estado nutricional inadequado,^(^^1^^)^ geralmente relacionado à gravidade da doença.^(^^2^^)^ A triagem nutricional é usada para detecção do risco nutricional e consiste em uma simples pesquisa para identificar indivíduos que necessitam de intervenção nutricional para reduzir o risco de complicações, infecção e mortalidade,^(^^3^^)^ diminuir a duração da hospitalização,^(^^4^^)^ melhorar a qualidade de vida e reduzir os custos dos cuidados de saúde.^(^^3^^)^ A intervenção nutricional fazen-se necessária em indivíduos com risco nutricional,^(^^2^^)^ para evitar desnutrição, que leva a taxas mais elevadas de morbidade e mortalidade, com pior prognóstico na recuperação de enfermidades subjacentes.^(^^3^^,^^5^^)^

O questionário *Nutritional Risk Screening 2002* (NRS 2002) é uma ferramenta de avaliação de aplicação rápida e fácil,^(^^5^^–^^7^^)^ que permite a detecção de alterações do estado nutricional e a determinação da necessidade de intervenção para manutenção ou recuperação do estado nutricional adequado em adultos e idosos.^(^^8^^)^ Entretanto, o NRS 2002 foi baseado em estudos com idosos de 70 anos ou mais, que recebem 1 ponto a mais no escore final como fator de adicional de risco.^(^^6^^)^ Essa ferramenta de avaliação foi desenvolvida em países europeus, onde adultos de 65 anos ou mais são denominados idosos. Segundo a Organização Mundial da Saúde (OMS), porém, a terceira idade tem início em idade inferior nos países em desenvolvimento, sendo considerados idosos aqueles com 60 anos ou mais de idade.^(^^8^^)^

Dado o impacto negativo da desnutrição em idosos, é importante conduzir estudos que adaptem esse método de triagem à população de idosos do Brasil, a fim de permitir a identificação precoce dos indivíduos que necessitam de intervenção nutricional para a manutenção ou recuperação do estado nutricional adequado.

## OBJETIVO

Avaliar o poder de discriminação diagnóstica de uma versão adaptada do *Nutritional Risk Screening 2002*, na qual o critério de idade para a soma de 1 ponto foi reduzido para 60 anos ou mais.

## MÉTODOS

Estudo transversal conduzido para testar o poder de discriminação diagnóstica de uma versão adaptada do NRS 2002. Pacientes de ambos os sexos com 60 anos ou mais e internados em enfermarias ou no setor cirúrgico de um hospital universitário localizado na cidade de Recife (PE), participaram do estudo. A coleta de dados foi realizada entre abril e dezembro de 2016, e todos os participantes assinaram um Termo de Consentimento Informado. Foram excluídos os pacientes inaptos para a avaliação antropométrica, os amputados, os que apresentavam edema ou ascite, os transplantados, os incapazes de fornecer informações e os que se encontravam em terapia nutricional enteral.

O risco nutricional foi avaliado nas primeiras 48 horas após a internação, por meio da administração de três questionários estruturados a cada paciente: NRS 2002 original, versão adaptada do NRS 2002 e *Mini Nutritional Assessment* (MNA) – este último o padrão-ouro.

A versão adaptada do NRS 2002 é uma modificação do modelo original proposto por Kondrup et al.,^(^^7^^)^ A diferença foi a redução do critério de idade para 60 anos para a adição de 1 ponto no escore final. A classificação de risco nutricional foi mantida, sendo o escore ≥3 considerado indicativo de risco.

O NRS 2002 original é composto de duas partes. A primeira parte aborda índice de massa corporal (IMC), perda de peso, alterações de ingestão alimentar e gravidade da doença de base. A segunda parte reserva-se aos pacientes que satisfazem um dos critérios da primeira parte e consiste em uma investigação mais profunda e na estratificação das variáveis avaliadas. No final do questionário, 1 ponto é somado para indivíduos com 70 anos de idade ou mais, sendo o risco nutricional definido como escore igual ou superior a 3 pontos.^(^^9^^)^

O MNA é tido como ferramenta padrão-ouro para avaliação nutricional em idosos e foi criado para investigar modificações de composição corporal, alterações recentes de peso, mudanças do padrão alimentar, grau de autonomia e autopercepção do estado de saúde nessa população.^(^^3^^)^ Esse questionário é capaz de predizer a morbidade, a mortalidade e os desfechos clínicos e contém 18 itens divididos em duas partes. A primeira possui seis itens de triagem, que geram um escore de até 14 pontos. Escore de 12 ou mais pontos indicam ausência de risco nutricional, sem necessidade de se completar o resto do questionário. Escores de 11 pontos ou menos indicam risco nutricional ou desnutrição, devendo o resto do questionário ser completado nesses casos. O MNA inclui dados antropométricos, avaliação global, dieta e autoavaliação subjetiva. O escore MNA total permite a classificação do risco nutricional da seguinte forma: <17 pontos para desnutrido, 17 a 23,5 pontos para risco de desnutrição e ≥ 24 pontos para bem nutrido.^(^^10^^)^

Variáveis demográficas e clínicas foram extraídas dos prontuários dos pacientes. As variáveis demográficas foram sexo (masculino ou feminino) e idade (60 a 69 anos ou ≥70 anos). As variáveis clínicas incluídas foram doença de base no momento da hospitalização (neoplasia, doença vascular, distúrbios geniturinários, neurológicos ou gastrintestinais, problemas como febre, anorexia, doença hepática, problemas respiratórios ou de nariz, ouvido e garganta, e hipóteses diagnósticas) e número de comorbidades (hipertensão arterial, *diabetes mellitus* e doença pulmonar obstrutiva crônica) associadas à doença de base (nenhuma, uma ou duas ou mais comorbidades).

As variáveis antropométricas consideradas foram IMC e circunferência do braço e da panturrilha. O IMC foi calculado como o peso (kg) dividido pela altura ao quadrado (m^2^). O peso (kg) foi aferido empregando-se uma balança analítica (Filizola, São Paulo, SP, Brasil) com capacidade máxima para 150kg e precisão de 100g. A altura foi determinada empregando-se um estadiômetro de alumínio acoplado à balança no plano horizontal de Frankfurt^(^^11^^)^ ou por meio da equação de Chumlea^(^^12^^)^ com base na altura do joelho. Os pontos de corte de IMC definidos por Lipschitz^(^^13^^)^ foram empregados na classificação do estado nutricional.

A circunferência do braço foi mensurada empregando-se uma fita métrica não elástica (comprimento máximo de 1,50m) posicionada no braço não dominante. Os participantes foram instruídos a fletir o braço em um ângulo de 90°, para facilitar a localização do ponto médio entre o acrômio e o olecrano, e, em seguida, a estender o braço ao longo do corpo, com a palma voltada para a coxa. O braço foi mensurado no ponto descrito, tendo-se o cuidado de evitar a compressão da pele e a folga da fita métrica.

A circunferência da panturrilha foi mensurada no membro inferior esquerdo, empregando-se uma fita métrica não elástica (cumprimento máximo de 1,50m) posicionada no ponto de maior diâmetro, com o paciente em posição supina ou sentada e com o joelho fletido em um ângulo de 90°.

Os programas (SPSS), versão 23.0 para Windows^®^, e Excel^®^ 2010 foram utilizados na análise estatística, e todos os testes foram aplicados com intervalo de confiança de 95% (IC95%). Os dados foram expressos como frequências absolutas e percentuais. As associações entre as variáveis categóricas foram testadas, empregando-se o teste χ^2^ de Pearson ou o teste exato de Fisher (quando o teste χ^2^ não fosse aplicável). A sensibilidade, a especificidade, a acurácia e os valores preditivos positivo e negativo foram determinados para as versões original e adaptada do NRS 2002 e comparados à MNA. O grau de concordância entre as ferramentas de avaliação foi determinado empregando-se a estatística Kappa. A margem de erro dos testes estatísticos foi de 5% (p<0,05) e IC95% foram calculados.

Este estudo foi aprovado pelo Comitê de Ética em Pesquisa em Seres Humanos do Centro de Ciências da Saúde da Universidade Federal de Pernambuco (UFPE), parecer 1.488.532, CAAE: 52561116.0.0000.5208.

## RESULTADOS

A amostra foi composta por 110 pacientes, 63,6% dos quais do sexo masculino. Pouco mais da metade (53,6%) tinha entre os 60 e 69 anos de idade, e 46,4% tinham 70 anos de idade ou mais (p=0,052). Os motivos de internação encontram-se listados na tabela 1, sendo os mais representados a neoplasia (28,2%) e a doença vascular (20,9%).

**Tabela 1:** Descrição da amostra (sexo e variáveis clínicas) de acordo com a faixa etária de idosos internados em um hospital universitário

Variável		Faixa etária			
		60 a 69 anos	70 ou mais	Grupo completo	Valor de p*
Sexo					0,857
	Masculino	38 (64,4)	32 (62,7)	70 (63,6)	
	Feminino	21 (35,6)	19 (37,3)	40 (36,4)	
Motivo de hospitalização					0,052†
	Neoplasia	16 (27,1)	15 (29,4)	31 (28,2)	
	Doença vascular	10 (16,9)	13 (25,5)	23 (20,9)	
	Geniturinária	4 (6,8)	10 (19,6)	14 (12,7)	
	Gastrintestinal	7 (11,9)	3 (5,9)	10 (9,1)	
	Neurológica	3 (5,1)	4 (7,8)	7 (6,4)	
	Outros‡	14 (23,7)	6 (11,8)	20 (18,2)	
	Não informado	5 (8,5)	0	5 (4,5)	
Comorbidade					0,372
	Inexistente	23 (39,0)	23 (45,1)	43 (39,1)	
	Uma doença	20 (33,9)	9 (17,6)	25 (22,7)	
	Duas ou mais doenças	16 (27,1)	19 (37,3)	42 (38,2)	

Resultados expressos como n (%).

* teste χ^2^ de Pearson;

† teste exato de Fisher;

‡ outras condições: febre, anorexia, doença hepática, distúrbios respiratórios, problemas de nariz, ouvido e garganta e hipóteses diagnósticas.

O risco nutricional estimado com base na versão adaptada do NRS 2002 ou no MNA não diferiu entre as faixas etárias. Em contrapartida, quando a versão original do NRS 2002 foi empregada, o risco nutricional foi mais frequente entre pacientes de 70 anos de idade ou mais (p=0,009) (Tabela 2).

**Tabela 2:** Risco nutricional estimado por meio de três métodos distintos, de acordo com a faixa etária de idosos internados em um hospital universitário

	NRS 2002 adaptado		NRS 2002		MNA	
Faixa etária, anos	n	Valor de p	n	Valor de p	n	Valor de p*
60-69	17	0,117	11	0,009*	27	0,116
≥70	22		21		31	

* teste χ^2^ de Pearson, p<0,05.

NRS: *Nutritional Risk Screening 2002*; MNA: *Mini Nutritional Assessment*.

Não houve associações significantes entre o risco nutricional estimado por meio das versões adaptada e original do NRS 2002 e doença ou comorbidades (Tabela 3). Entretanto, foram observadas associações significantes entre risco nutricional e tipo de doença (p=0,019) e risco nutricional e comorbidades (p=0,022) quando o risco foi estimado com base no MNA (dados não apresentados na tabela).

**Tabela 3:** Associações entre risco nutricional estimado com base nas versões adaptada e original do *Nutritional Risk Screening* 2002 e motivos de internação/ comorbidades em idosos internados em um hospital universitário

		NRS 2002 adaptado			NRS 2002		
Variável		Sim	Não	Valor de p	Sim	Não	Valor de p
Motivo de admissão				0,419*			0,591*
	Neoplasia	10 (32,3)	21 (67,7)		10 (32,3)	21 (67,7)	
	Doença Vascular	10 (43,5)	13 (56,5)		7 (30,4)	16 (69,6)	
	Geniturinário	4 (28,6)	10 (71,4)		4 (28,6)	10 (71,4)	
	Gastrintestinal	3 (30,0)	7 (70,0)		2 (20,0)	8 (80,0)	
	Neurológico	5 (71,4)	2 (28,6)		4 (57,1)	3 (42,9)	
	Outros†	6 (30,0)	14 (70,0)		4 (20,0)	16 (80,0)	
Comorbidade				0,216			0,350
	Inexistente	18 (42,9)	24 (57,1)		12 (28,6)	30 (71,4)	
	Uma doença	11 (25,6)	32 (74,4)		10 (23,3)	33 (7,7)	
	Duas ou mais	10 (40,0)	15 (60,0)		10 (40,0)	15 (60,0)	
	doenças						

Resultados expressos como n (%).

* teste χ^2^ de Pearson;

† teste exato de Fisher.

NRS: *Nutritional Risk Screening 2002*.

A tabela 4 mostra a frequência do risco nutricional estimada com base no MNA e nas versões original e adaptada do NRS 2002 em pacientes de 60 anos ou mais. A frequência mais alta foi encontrada quando a ferramenta de avaliação foi o MNA, seguida da versão adaptada do NRS 2002.

**Tabela 4:** Frequência de risco nutricional entre pacientes de idade igual ou superior a 60 anos (n=110), de acordo com a ferramenta de triagem nutricional

Ferramenta de triagem	Risco nutricional	Sem risco nutricional
MNA	52,7	47,3
NRS 2002 original	29,1	70,9
NRS 2002 adaptado	35,5	64,5

Resultados expressos como %.

MNA: *Mini Nutritional Assessment*; NRS 2002: *Nutritional Risk Screening 2002*.

A tabela 5 mostra os resultados da análise de concordância entre os métodos, tomando-se o MNA como padrão-ouro. A versão adaptada do NRS 2002 mostrou-se mais sensível do que a original (60,3% e 50%, respectivamente), embora ainda classificada como insuficiente. A sensibilidade e a especificidade total das versões original e adaptada do NRS 2002 foram de 144,2 e 152,6, respectivamente.

**Tabela 5:** Acurácia diagnóstica e intervalos de confiança de 95% das versões adaptada e original do *Nutritional Risk Screening 2002* comparados ao *Mini Nutritional Assessment* (padrão-ouro)

Instrumento	Sensibilidade	Especificidade	VPP	VPN	Acurácia
	(n1/n)	(n1/n)	(n1/n)	(n1/n)	(n1/n)
NRS 2002 adaptado	60,3 (35/58)	92,3 (48,52)	89,7 (35/39)	67,6 (48/71)	75,5 (83/110)
NRS 2002 original	50,0 (29/58)	94,2 (49,52)	90,6 (29/32)	62,8 (49/78)	70,9 (78/110)

VPP: valor preditivo positivo; VNP: valor preditivo negativo; NRS 2002: *Nutritional Risk Screening 2002*.

## DISCUSSÃO

Os resultados obtidos por meio da versão adaptada do NRS 2002 usada neste estudo refletem os achados derivados da versão original do questionário relatados na literatura, mostrando maior especificidade do que sensibilidade e indicando boa capacidade de identificação de pacientes sem risco nutricional. Em estudo envolvendo idosos de Genebra e Berlim, Kyle et al.,^(^^14^^)^ encontraram índice de risco nutricional de 28% empregando o NRS 2002, com sensibilidade, especificidade, valores preditivos positivo e negativo de 62%, 93%, 85% e 79%, respectivamente – valores esses superiores aos obtidos com a ferramenta *Malnutrition Universal Screening Tool* e relatados na *NR Initiative*.

Entretanto, conforme a sensibilidade aumenta, o número de falsos-positivos também aumenta, e, da mesma forma, o aumento da especificidade leva à elevação no número de falsos-negativos.^(^^15^^)^ Apesar do grau equivalente de concordância entre o MNA e as duas versões do NRS 2002 (concordância moderada de acordo com o coeficiente Kappa), a versão adaptada mostrou-se descritivamente mais eficiente, devido à maior sensibilidade. Outro impacto positivo da adaptação do NRS 2002 foi a maior sensibilidade e especificidade total em relação à versão original – mais um fator que corrobora o maior poder de discriminação diagnóstica da versão adaptada em relação à original.

Quanto aos principais motivos de internação hospitalar, os achados deste estudo reforçam dados de pesquisa conduzida por Bezerra et al.,^(^^5^^)^ em uma policlínica localizada na cidade de Natal (RN), na qual a maioria dos pacientes (26,9%) sofria de câncer, problemas geniturinários (11,5%), distúrbios respiratórios (11,5%) e distúrbios gastrintestinais (7,7%). Em contrapartida, assim como em países desenvolvidos, os principais diagnósticos encontrados em um serviço privado de saúde localizado na cidade de Porto Alegre (RS) foram distúrbios musculoesqueléticos (22,2%), doenças respiratórias (11,1%) e doenças cardiovasculares (11,1%).^(^^16^^)^ Koren-Hakim et al.,^(^^17^^)^ relataram hipertensão (69,3%), doença cardiovascular (52,1%) e distúrbios musculoesqueléticos (42,3%) como os principais motivos de hospitalização. Diferenças nas características das amostras podem ser explicadas pela disparidade de classe econômica entre as populações estudadas, dada a associação entre maior poder aquisitivo e melhor estado nutricional.

Este estudo não apontou associações significantes entre doença e risco nutricional estimado com base nas duas versões do NRS 2002. Isso pode ser devido ao fato de a maioria dos pacientes se encontrar em estágios iniciais da doença e ter sido internada para diagnóstico da doença de base. Cabe destacar que, diferentemente de outras ferramentas de triagem nutricional, o MNA não leva em conta a doença de base, a qual influencia na determinação de associações entre motivo de hospitalização e risco nutricional.

A frequência de risco nutricional estimado por meio das versões original e adaptada do NRS 2002 não diferiu entre pacientes com neoplasia. Achados semelhantes foram relatados por Lisboa da Silva et al.,^(^^18^^)^ em amostra de pacientes exclusivamente clínicos atendidos no mesmo hospital universitário e avaliados por meio da versão original do NRS 2002 (índice de 38,4% de risco nutricional em pacientes com câncer).

Estudos envolvendo triagem nutricional baseiam-se em métodos de detecção de risco nutricional e medidas distintos, além de pacientes com diagnósticos diversos. A frequência de risco nutricional foi mais baixa neste estudo do que em ambulatório cirúrgico (69,3%)^(^^19^^)^ e na clínica médica (51,3%) de um hospital universitário localizado na cidade de Recife.^(^^18^^)^ Essa disparidade pode ser explicada por diferenças relacionadas a especialidades clínicas e doenças entre as amostras.

No que se refere à detecção do risco nutricional, índices semelhantes foram encontrados nas diferentes faixas etárias (60 a 69 anos e acima de 70 anos) quando estimados por meio da versão adaptada do NRS 2002 e do MNA. Entretanto, índices significativamente mais altos de risco nutricional foram encontrados em indivíduos de 70 anos ou mais, avaliados por meio da versão original do NRS 2002, sugerindo que esse último questionário não é adequado para a triagem de indivíduos de 60 a 69 anos

Estudos investigando a concordância entre o NRS 2002 e o MNA são escassos. Em um estudo conduzido por Neelemaat et al.,^(^^20^^)^ com idosos de 60 anos ou mais na cidade de Amsterdã, na Holanda, o NRS 2002 mostrou sensibilidade (92%), especificidade (83%), valor preditivo positivo (70%) e valor preditivo negativo (96%) descritivamente mais elevados do que os obtidos neste estudo. Entretanto, os autores empregaram medidas antropométricas como padrão-ouro, sendo que essa não é a melhor ferramenta de triagem nutricional para idosos. Além disso, o poder de discriminação diagnóstica do método usado neste estudo foi determinado com base na estatística Kappa, o que permite maior acurácia na comparação com o padrão-ouro.

Este estudo tem limitações dignas de nota. O tamanho amostral foi pequeno para ser representativo. Além disso, sendo o desenho experimental transversal, o desfecho é considerado em um momento específico, o que impede a determinação de relações potenciais entre causa e efeito.

## CONCLUSÃO

Os resultados aqui apresentados sugerem que a versão adaptada do *Nutritional Risk Screening 2002* para idosos de 60 anos ou mais tem maior poder de discriminação diagnóstica do que a versão original, permitindo a detecção mais eficiente do risco nutricional em idosos hospitalizados. Entretanto, novos estudos fazem-se necessários para confirmar tais achados.
